# How to induce defense responses in wild plant populations? Using bilberry (*Vaccinium myrtillus*) as example

**DOI:** 10.1002/ece3.2687

**Published:** 2017-02-15

**Authors:** Tarald Seldal, Stein Joar Hegland, Knut Rydgren, Cesar Rodriguez‐Saona, Joachim Paul Töpper

**Affiliations:** ^1^ Faculty of Engineering and ScienceSogn & Fjordane University CollegeSogndalNorway; ^2^ Department of EntomologyRutgers UniversityChatsworthNJUSA; ^3^ Norwegian Institute of Nature ResearchBergenNorway

**Keywords:** bilberry, chemical elicitor, clipping experiment, deer, field experiment, growth, herbivory, insects, methyljasmonate, Vaccinium

## Abstract

Inducible plant defense is a beneficial strategy for plants, which imply that plants should allocate resources from growth and reproduction to defense when herbivores attack. Plant ecologist has often studied defense responses in wild populations by biomass clipping experiments, whereas laboratory and greenhouse experiments in addition apply chemical elicitors to induce defense responses. To investigate whether field ecologists could benefit from methods used in laboratory and greenhouse studies, we established a randomized block‐design in a pine‐bilberry forest in Western Norway. We tested whether we could activate defense responses in bilberry (*Vaccinium myrtillus*) by nine different treatments using clipping (leaf tissue or branch removal) with or without chemical treatment by methyljasmonate (MeJA). We subsequently measured consequences of induced defenses through vegetative growth and insect herbivory during one growing season. Our results showed that only MeJA‐treated plants showed consistent defense responses through suppressed vegetative growth and reduced herbivory by leaf‐chewing insects, suggesting an allocation of resources from growth to defense. Leaf tissue removal reduced insect herbivory equal to the effect of the MeJa treatments, but had no negative impact on growth. Branch removal did not reduce insect herbivory or vegetative growth. MeJa treatment and clipping combined did not give an additional defense response. In this study, we investigated how to induce defense responses in wild plant populations under natural field conditions. Our results show that using the chemical elicitor MeJA, with or without biomass clipping, may be a better method to induce defense response in field experiments than clipping of leaves or branches that often has been used in ecological field studies.

## 1 Introduction

Plant defense theory predicts that plants under attack by herbivores should divert resources from growth and reproduction to defense, but when the attack has passed, they should once again allocate more resources to growth and reproduction (Agrawal, 2011; Agrawal, Conner, & Rasman, 2010; Cipollini & Heil, 2010; Sampedro, Moreira, & Zas, 2011). Plants incur costs if limiting resources such as nitrogen or carbon are invested in defense (Redman, Cipollini, & Schultz, 2001) as activation of plant defense genes is normally followed by a downregulation of genes important to photosynthesis (Halitschke, Hamilton, & Kessler, 2011; Heidel & Baldwin, 2004; Nabity, Zavala, & DeLucia, 2013).

Induced plant defense systems rely on a complex signaling and regulatory network of plant hormones where jasmonic acid and its derivative methyl jasmonate (MeJa) are important elicitors of plant defense systems against leaf‐chewing insects and necrotrophic pathogens (Moreira, Zas, & Sampedro, 2012; Pieterse, Van der Does, Zamioudis, Leon‐Reyes, & Van Wees, 2012). As a consequence, plant defense systems can be activated experimentally by exogenous application of MeJa (Cipollini, Mbagwu, Barto, Hillstrom, & Enright, 2005; Heijari et al., 2005; Moreira, Sampedro, & Zas, 2009; Moreira et al., 2012), a ubiquitous defense signal in plants released in response to tissue damages (Koo & Howe, 2009; Pieterse et al., 2012). Several studies have shown that experimental induction of plant defense systems by exogenously applied MeJa causes consequences of induced defenses such as reduced growth and seed production (Accamando & Cronin, 2012; Baldwin, 1998; Cipollini & Heil, 2010; Corrado et al., 2011; Hegland, Seldal, Lilleeng, & Rydgren, 2016; Nabity et al., 2013).

Studies of inducible plant defense systems have mainly been accomplished under controlled laboratory or greenhouse conditions or in field studies with crops (Howe, 2004; Howe & Jander, 2008; Moreira et al., 2012; but see Hegland et al., 2016). Therefore, we need more knowledge of how plant defense systems function in wild populations under natural field conditions and what the ecological consequences may be through cascading effects onto other trophic levels (Karban, Yang, & Edwards, 2014; Rodriguez‐Saona, Mescher, & De Moraes, 2013). In field studies performed to estimate ecological consequences, clipping has often been used to simulate herbivory followed by analyses of plant nutritional quality and leaf palatability (Nordin, Strengbom, Witzell, Näsholm, & Ericson, 2005; Pato & Obeso, 2012, 2013; Strengbom, Olofsson, Witzell, & Dahlgren, 2003). It is well known, however, that herbivore‐specific cues transmitted from the herbivores saliva to the site of the tissue damage, or other stress‐related cues, are required to fully activate the plant`s chemical defense system (Howe, 2004; Howe & Jander, 2008; Parè & Tumlinson, 1997; Turlings, Tumlinson, & Lewis, 1990).

In boreal forests, bilberry (*Vaccinium myrtillus* L.) is a dominant evergreen dwarf shrub and an important food source for insects and vertebrate herbivores, pollinators, and fruit‐eating birds and mammals (Hjälten, Danell, & Ericson, 2004; Jacquemart, 1993; Selås, 2001; Wegge, Olstad, Gregersen, Hjeljord, & Sivkov, 2005; Welch, Keay, Kendall, & Robbins, 1997). Bilberry is thus a suitable study plant to test whether we could activate defense responses under natural field condition and to estimate ecological consequences. We established a field experiment in wild populations of bilberry and tested how growth and defense of plants responded to combinations of exogenously applied MeJa and clipping (leaf tissue removal and branch removal). As exogenously applied MeJa normally gives defense responses comparable to those obtained in response to attack by herbivores or pathogens (Moreira et al., 2012; Pieterse et al., 2012), we predicted that both MeJa application and clipping would reduce subsequent attack by leaf‐chewing insects (prediction I; Fürstenberg‐Hägg, Zagrobelny, & Bak, 2013; Moreira et al., 2012; Rodriguez‐Saona, Polashock, & Malo, 2013). As inducible plant defense is expected to involve an allocation of resources from growth to defense (Agrawal et al., 2010; Sampedro et al., 2011), we predicted reduced growth of both MeJa‐treated plants and plants exposed to clipping (prediction II). Furthermore, as plants under herbivore attack can respond differently from plants exposed to mechanical tissue damages (Koo & Howe, 2009; Moreira et al., 2012), we predicted that the effects on vegetative growth and the reduction in herbivore attacks should be stronger for MeJa‐treated plants than for plants exposed to clipping (prediction III) (Karban et al., 2014; Moreira et al., 2012). Studies of other *Vaccinium* species under controlled laboratory conditions have shown that plant defense systems can be activated in response to mechanical tissue removal, natural herbivory, or by the use of exogenously applied elicitors of plant defense systems such as MeJa (Rodriguez‐Saona, Polashock et al., 2013). Therefore, we predicted that MeJa treatment and clipping combined would give the strongest suppression of vegetative growth and reduction in subsequent attack by leaf‐chewing insects as compared to each treatment alone (prediction IV). Testing these predictions could give results that might guide ecologists and plant biologist on how to induce defense responses in plants under natural conditions.

## 2 Materials and Methods

### 2.1 Study site and species

We conducted our study in a pine–bilberry forest in Sogndal, Western Norway, at 150–200 m above sea level. The understory was dominated by bilberry and lingonberry (*Vaccinium vitis‐idaea* L.), graminoids and bryophytes, and is located in the southern boreal zone with an annual precipitation of 700–900 mm and a mean summer temperature of 12–16°C (Moen, 1999). Bilberry is a long‐lived deciduous, rhizomatous shrub with aerial erect shoots, usually 10–60 cm high (Flower‐Ellis, 1971; Ritchie, 1956). The species is known to be fairly herbivore tolerant because of its extended clonal growth and high regrowth ability (Dahlgren, Oksanen, Sjödin, & Olofsson, 2007; Hegland, Jongejans, & Rydgren, 2010; Tolvanen, Laine, Pakonen, Saari, & Havas, 1994).

### 2.2 Experimental design

In June 2012, we established fifteen 10 × 10 m blocks, leaving minimum 10 m between each block, within a 0.5 km^2^ area that had high cover of bilberry (ca. >25%). Within each block, nine bilberry ramets, ranging from 10 to 25 cm in height, were randomly selected and individually marked and exposed to the nine different treatments (Table 1). To reduce the possibility for plant–plant communication via airborne defense volatiles emitted from treated neighbor plants (Karban et al., 2014; Rodriguez‐Saona, Mescher et al., 2013), the selected ramets was >3.5 m apart. This distance also reduces the probability for clonal connections between ramets in bilberry (Albert, Raspé, & Jacquemart, 2003; Albert, Raspè, & Jacquemart, 2004; Flower‐Ellis, 1971).

**Table 1:** Experimental treatments of bilberry (*Vaccinium myrtillus*) ramets. Each treatment was repeated three times over a 2‐week period

Group	Treatment
Control	Exogenous spraying with the control solution
MeJa‐5	Exogenous spraying with 5 mM MeJa
MeJa‐10	Exogenous spraying with 10 mM MeJa
LTR‐0	Leaf tissue removal + exogenous spraying with the control solution
LTR‐5	Leaf tissue removal + 5 mM exogenous spraying with MeJa
LTR‐10	Leaf tissue removal + 10 mM exogenous spraying with MeJa
BR‐0	Branch removal + exogenous spraying with the control solution
BR‐5	Branch removal + 5 mM exogenous spraying with MeJa
BR‐10	Branch removal + 10 mM exogenous spraying with MeJa

MeJa, methyljasmonate; LTR, leaf tissue removal; BR, branch removal.

### 2.3 Experimental treatments and sampling procedures

**Figure 1:**
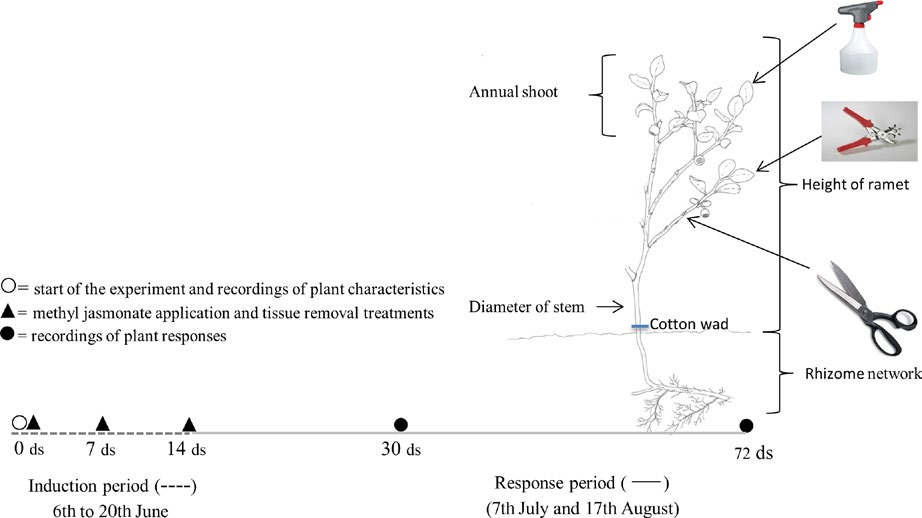
Drawing of study species including the experimental design with treatments, size recordings, and timeline for the induction and response periods

We started the chemical induction treatments on 6 June 2012 by spraying the ramets with either 5 or 10 mM MeJa (Bedoukian Research, Danbury, CT, USA), or a control solution of water and ethanol until the point of runoff (Figure 1; Table 1). To achieve the desired concentrations of MeJa, we diluted MeJa 1:10 with 95% (v/v) ethanol and rediluted the solutions with water to get 5 and 10 mM MeJa. To avoid rapid evaporation of MeJa, we attached a cotton wad to the stem at the ground saturated with either 5 or 10 mM MeJa or the control solution. Spraying was repeated three times with 1‐week intervals (Figure 1).

To separate the effects of MeJa treatments from the clipping treatments, we removed leaf tissue (holes in leaves) and annual shoots (here also called branch) at the selected ramets three times in a 2‐week period. Insect herbivory was simulated by removing two circles, 2 mm in diameter, from each of the five lower leaves once a week over the treatment period (Figure 1; Table 1). We simulated ungulate herbivory by removing one annual shoot (branch) once a week over the treatment period from each of the ramets. At the end of the 2‐week treatment period, three annual shoots had been removed from each of the ramets exposed to simulated ungulate herbivory, whereas 15 leaves, with two holes in each leaf, were damaged on ramets exposed to simulated insect herbivory. In sum, this resulted in nine different combinations of the Meja‐ and herbivory treatments, including the control, to mimic natural herbivory (Table 1). After the treatment period, we removed the cotton wad from all of the ramets. Prior to the experimental treatments (6 June), we recorded plant height, number of shoots, stem diameter, number of flowers, number of berries, total number of leaves, and number of leaves grazed by leaf‐chewing insects for each of the 135 ramets (15 blocks × 9 plants per block). These recordings were repeated twice, 30 (7 July) and 72 days (17 August) after the start of the treatments (Figure 1). The experiment differs from Hegland et al. (2016) that only applied 10 mM MeJa solution to a 0.5 × 0.5 m plot level to study defense responses and that did not compare different types of treatments.

As many insects react rapidly to disturbances and drop to the ground to hide and avoid predation (Ohno & Miyatake, 2007 and references therein)), direct records of insect species and abundance are difficult in field studies and may even result in unreliable estimates. Such avoidance behavior has been observed in the study area for the most abundant insect herbivore on bilberry, *Geometridae* caterpillars (Seldal T, personal observation). Quantifying leaf tissue consumption by the leaf area removed by leaf‐chewing insects was not considered as an appropriate field method as it would have been too time‐consuming and also could stress the plants considerably. Therefore, we counted the number of leaves per plant that had chewing marks as a proxy for the abundance of leaf‐chewing insects and as an indicator of bilberry defense. This is a simple and cost‐efficient method, often applied in field studies where the objective is to study possible changes in leaf palatability by leaf‐chewing insects in response to experimental treatments (Hegland, Rydgren, & Seldal, 2005; Hegland et al., 2016; Pato & Obeso, 2012, 2013), and it is also known as a common consequence of induced defenses in laboratory experiments on induced defense in *Vaccinium* species (Rodriguez‐Saona, Mescher et al., 2013; Rodriguez‐Saona, Polashock et al., 2013; Rodriguez‐Saona, Rodriguez‐Saona, & Frost, 2009). The size measurements (stem diameter, plant height and number of annual shoots) of bilberry were used to estimate the dry mass nondestructively (in log_2_ units) of the ramets from in situ morphological measurements at each census based on the biomass formula developed for bilberry by Hegland et al. (2010). By following the responses of individually marked plants during the growing season, we could record changes in the amount of herbivory by leaf‐chewing insects and changes in plant growth in response to the different treatments. These changes were used to analyze the hypothesized trade‐off between growth and defense in bilberry (see Section 2.4).

### 2.4 Data analysis

We examined whether exogenous MeJa treatments and/or mechanical tissue removal reduced the attack by leaf‐chewing insects (i.e., reduced subsequent insect herbivory) and caused changes in resource allocation from plant growth to defense (i.e., reduced vegetative growth). Therefore, we tested how the increase in the proportion of insect‐grazed leaves and plant biomass changed from June to August in response to the treatments applied in early June. As there were few flowers on the plants in the study area, probably due to shading effects of trees and annual variation (personal observation), treatment effects on flowering frequency and berry production could not be tested. Twenty‐three individual ramets with <2/5 of their leaf or shoot tissue untreated (i.e., <25 leaves before clipping holes in 15 of them, and <5 shoots before removing three) were removed from the dataset prior to the statistical analysis to avoid potential bias from severe experimental (clipping) damage. As also excessive loss of leaves between registrations constitutes a potential bias (as we don't know whether or not these leaves were insect grazed and why they were lost), we removed 11 individuals which lost more than 1/5 of their leaves during the experiment. Six more individuals were entirely lost during the experiment, most likely due to ungulate grazing, resulting in a final sample size of 94 independent individual ramets for analysis.

We used linear mixed effects models with Gaussian error distribution and identity link for dry mass and generalized linear mixed effect models with binominal error distribution and logit link for ratio of insect‐grazed leaves. Time (numerical), MeJa concentration (categorical; three levels: 0, 5 and 10 mM), and clipping (categorical; three levels: control, leaf tissue removal, and branch removal) as well as their interactions were used as fixed effects predictors. The nested spatial structure of ramets within block were used as random effects to account for both dependencies within blocks and ramets and variability in size and grazing pressure at the start of the experiment. We allowed for both random intercepts and slopes (i.e., time) for ramet and block. Random slopes and fixed effects elements were removed from the models when not contributing significantly to model performance in a backwards selection procedure using likelihood ratio tests. All statistical analyses were performed in R version 2.15.0 (R Development Core Team 2012) with the packages “lme4” for linear mixed effects models (Bates, Maechler, & Bolker, 2012) and “language R” for Markov Chain Monte Carlo sampling, last of which was applied to the models with Gaussian error distributions to obtain conservative *p*‐values (Baayen, 2011).

## 3 Results

**Figure 2:**
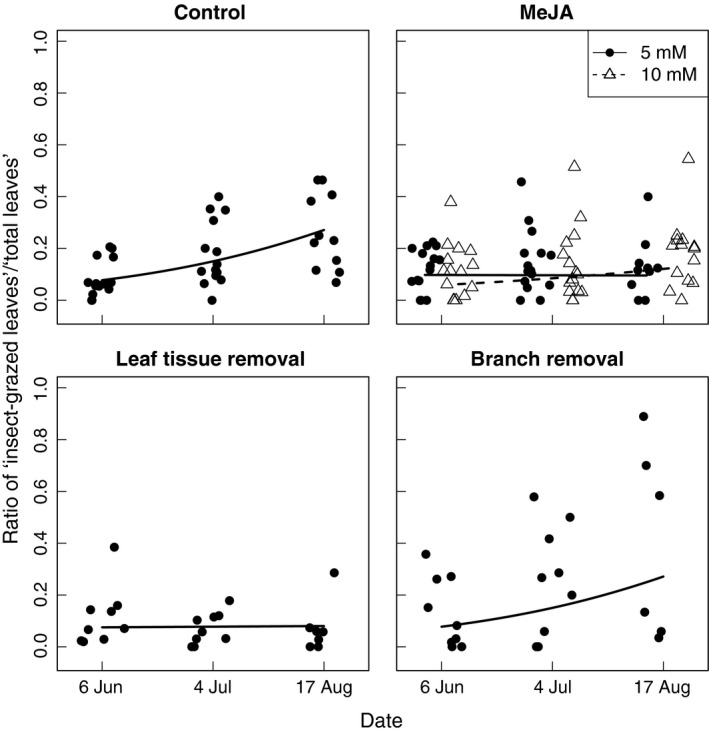
Development of the proportion of insect‐grazed bilberry (*Vaccinium myrtillus*) leaves from June to August for the control treatment, the methyljasmonate (MeJa) treatments, and the clipping treatments. Data points were jittered around the three dates in order to promote readability of the plot. Lines indicate the mixed effects models predictions for the respective treatments

**Figure 3:**
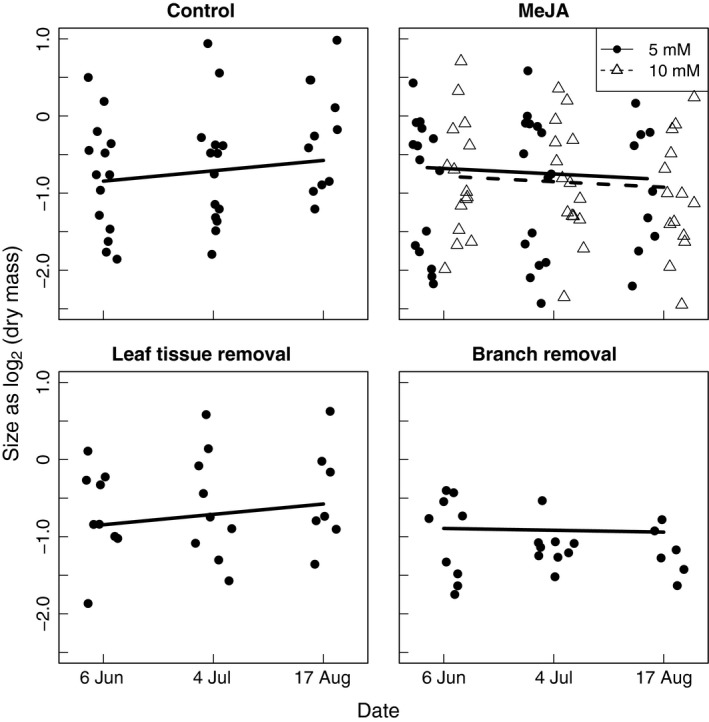
Bilberry (*Vaccinium myrtillus*) growth (dry mass) from June to August for the control treatment, the methyljasmonate (MeJa) treatments, and the clipping treatments. Data points were jittered around the three dates in order to promote readability of the plot. Lines indicate the mixed effects models predictions for the respective treatments

For control plants and plants exposed to branch removal, leaf‐chewing insects increased their consumption of leaf tissue from June to August, whereas herbivory was significantly reduced for MeJa‐treated plants and plants exposed to leaf tissue removal (Figure 2; Table 2; supporting prediction I). Also, biomass increased continuously for control plants and plants exposed to tissue removal treatments through the trial period, whereas biomass was significantly reduced for MeJa‐treated plants. The reduction in biomass of MeJa‐treated plants was largely due to reduced plant height and stem diameter, suggesting an allocation of resources from growth to defense (Figure 3; Table 2; supporting prediction II). Only MeJa‐treated plants showed both reduced growth and reduced insect herbivory, indicating an allocation of resources from growth to defense (Table 2; supporting prediction III). MeJa treatments and clipping treatments combined did not give an additional reduction in vegetative growth or herbivory by leaf‐chewing insects (Table 2; not supporting prediction IV).

**Table 2:** Effects of methyljasmonate (MeJa) treatment and/or clipping treatments on growth and insect herbivory in bilberry (*Vaccinium myrtillus*)

	Insect‐grazed leaves		Dry mass	
	Coef.	*SE*	Coef.	*SE*
Control	0.74	0.09***	0.10	0.04
∆ MeJa (5 mM)	−0.76	0.13***	−0.17	0.05**
∆ MeJa (10 mM)	−0.35	0.13***	−0.17	0.05**
∆ LTR	−0.79	0.18***	NA	NA
∆ BR	NA	NA	−0.14	0.05
∆ MeJa (5 mM) + LTR	1.24	0.24***	NA	NA
∆ MeJa (5 mM) + BR	NA	NA	NA	NA
∆ MeJa (10 mM) + LTR	0.44	0.47 (*)	NA	NA
∆ MeJa (10 mM) + BR	NA	NA	NA	NA

Given are the control time slopes for the mixed effects models on the ratio of insect‐grazed leaves and dry mass (plant growth) and the differences in time slopes for the different treatments (Coef.) together with standard errors (*SE*). MeJa = methyljasmonate, LTR = leaf tissue removal, BR = branch removal. Significance is indicated by an asterisk (Significance codes: ***<0.001, **<0.01, *<0.05, (*) <0.1), whereas “NA” indicate nonsignificant parameters that were removed from the model. Main effect coefficients (light gray rows) are given as differences (∆) from the control (white row), and interaction coefficients (dark gray rows) are given as differences (∆) from the added effects of the respective main effects.

The variability issued from the spatial structure of the experiment was considerable between ramets, but smaller among blocks as indicated by the random effects of the respective models (Table 3). For plant biomass, the intercepts and slopes varied greatly among ramets, meaning that the sampled plants covered a substantial range of sizes at the start of the experiment (intercepts) and showed differences in growth (slopes). The intercepts of ramets and blocks varied also for the proportion of insect‐grazed leaves, showing a substantial variation in previous herbivory among both ramets and blocks prior to the start of the experiment.

**Table 3:** Variability issued from the spatial structure of the experiment as indicated by the random effects of the mixed effects models

	Insect‐grazed leaves	Dry mass
Intercept (ramet:block)	0.93	0.64
Time (ramet:block)	NA	0.17
Intercept (block)	0.41	0.22
Time (block)	NA	NA
Fixed effect for intercept	−3.22	−0.92
Fixed effect for time	0.74	0.10

Random effects on intercepts indicate variability in dry mass and grazing intensity between blocks and ramets‐within‐blocks at the start of the experiment. Random effects on the time slopes (here only for dry mass) indicate variability in growth during the experiment. Random effects are given as standard deviations of fixed effects intercept and fixed main effect of time across ramets and blocks. For comparison, these fixed effects coefficients are shown in the two bottom rows. NA's indicate parameters that did not contribute significantly to the respective model and hence were removed.

## 4 Discussion

In this study, we showed that exogenously applied MeJa elicits a defense response in a wild bilberry population at the expense of plant growth. This study was specifically designed to test various treatment methods, both physically and chemically, in order to induce defense responses under natural field conditions. In contrast to laboratory and greenhouse studies or studies in agricultural systems, ecological field studies have to deal with considerable variation in growth conditions, plant age, genetical composition, pathogenic infestation, and previous herbivory. This variation is also of ecological importance as variation is symptomatic of how nature works. Moreover, in field studies of plant defense responses, precipitation, evaporation, and wind can dilute the concentration of airborne plant defense volatiles and thereby reduce their repellent role in plant–herbivore interactions. Nevertheless, our study showed that leaf‐chewing insects reduced their attack on MeJa‐treated plants, which supported our prediction I. These findings are consistent with a field experiment testing winter browsing effects on bilberry only applying chemical treatment by MeJa (Hegland et al., 2016), and results from controlled laboratory studies of other *Vaccinium* species where exogenously applied MeJa have been found to activate defense genes and protect the plants from subsequent insect attacks (Rodriguez‐Saona, Mescher et al., 2013; Rodriguez‐Saona, Polashock et al., 2013). Exogenously applied MeJa have been reported to reduce the colonization rate, survival, and reproduction of the spruce bark beetle (*Ips typographus*) on Norway spruce (*Picea abies*) (Erbilgin, Krokene, Christiansen, Zeneli, & Gershenzon, 2006). Moreover, Heijari, Nerg, Kainulainen, Vuorinen, and Holopainen (2008) showed that MeJa application to Scots pine (*Pinus sylvestris* L.) reduced the growth rates of sawflies, whereas Rodriguez‐Saona, Polashock et al. (2013) found that gypsy moth (*Lymantria dispar*) herbivory, mechanical tissue damage, and exogenously applied MeJa activated plant defense genes and decreased caterpillar attack on American cranberry (*Vaccinium macrocarpon*). Our study adds to this growing body of evidence by showing that boreal plants under natural conditions may have their defense system triggered by MeJa which reduces the herbivory on bilberry leaves by leaf‐chewing caterpillars.

The result that only MeJa‐treated plants appeared to divert resources from growth to defense during the growing season was consistent with our prediction II. Shifts in resource allocation from growth to defense in MeJa‐treated plants has been reported for wild tobacco (Nabity et al., 2013) and pine trees growing on nutrient deficient soil (Sampedro et al., 2011). In a field study in Canada, Percival and MacKenzie (2007) reported that MeJa‐treated blueberry plants (*Vaccinium angustifolium*) significantly reduced their berry production over two succeeding years, probably caused by an allocation of resources from growth and reproduction to defense. In contrast to MeJa‐treated plants, the clipping treatments (branch removal or leaf tissue removal) alone did not suppress plant growth, suggesting that there was no allocation of resources from growth to defense following such clipping treatments. The leaf tissue removal, however, did reduce attack by leaf‐chewing insects similar to the reduction in insect herbivory observed for MeJa‐treated plants (Table 2). Insect herbivores may simply have avoided these plants because the holes in the leaves mimic previous insect attacks and reduce the available biomass, indicating a possible drawback with this physical method. More likely repeated tissue removal may have induced a defense response sufficient to reduce subsequent insect attacks, but too weak to suppress plant growth. As the activation of plant defense systems involves cues not induced by mechanical tissue damage such as herbivore movement pattern, feeding mode, and cues from the herbivores saliva (Howe & Jander, 2008; Parè & Tumlinson, 1997; Turlings et al., 1990), it is likely that clipping alone was apparently not sufficient to activate a strong defense response in bilberry at the expense of growth under natural field conditions. The branch removal treatment had no negative effect on subsequent herbivory or plant growth. Thus, only MeJa treatments resulted in a negative effect on both subsequent insect herbivory and plant growth consistent with prediction III and the expected trade‐off that will appear when complete systemic induced plant defense is activated. This conclusion was supported by the fact that there was no additional reduction in insect herbivory or plant growth by combining the MeJa treatments with mechanical clipping as we first expected (i.e., prediction IV).

The activation of plant defense systems by both exogenously applied MeJa and mechanical tissue removal will clearly differ from the activation caused by herbivores (Koo & Howe, 2009; Moreira et al., 2012). Our study show that studies in wild plant population may rather use exogenous application of MeJa than clipping experiments and may not even need to combine MeJA treatment with mechanical tissue removal to activate the defense system. Furthermore, clipping alone was not sufficient to induce a trade‐off between growth and defense, under natural field conditions with considerable variation in plants and environment. Such trade‐off is the fundament for a strong and complete systemic defense response in plants. The results and conclusions from many clipping experiments should therefore be evaluated with this new knowledge in mind. Our study highlights the need to experimentally activate plant defense systems under natural field conditions by the use of chemical elicitors of plant defense systems.

## Conflict of Interest

None declared.
